# Determination of Parameters for an Entropy-Based Atrial Fibrillation Detector

**DOI:** 10.3390/e23091199

**Published:** 2021-09-11

**Authors:** Lina Zhao, Jianqing Li, Xiangkui Wan, Shoushui Wei, Chengyu Liu

**Affiliations:** 1 School of Instrument Science and Engineering, Southeast University, Nanjing 210096, China; 101102013@seu.edu.cn; 2 Hubei Collaborative Innovation Center for High-Efficiency Utilization of Solar Energy, Hubei University of Technology, Wuhan 430068, China; wanxiangkui@163.com; 3 School of Control Science and Engineering, Shandong University, Jinan 250061, China; sswei@sdu.edu.cn

**Keywords:** entropy measure, atrial fibrillation (AF), heart rate variability, entropy-based AF detector

## Abstract

Entropy algorithm is an important nonlinear method for cardiovascular disease detection due to its power in analyzing short-term time series. In previous a study, we proposed a new entropy-based atrial fibrillation (AF) detector, i.e., Entropy_AF_, which showed a high classification accuracy in identifying AF and non-AF rhythms. As a variation of entropy measures, Entropy_AF_ has two parameters that need to be initialized before the calculation: (1) tolerance threshold *r* and (2) similarity weight *n*. In this study, a comprehensive analysis for the two parameters determination was presented, aiming to achieve a high detection accuracy for AF events. Data were from the MIT-BIH AF database. RR interval recordings were segmented using a 30-beat time window. The parameters *r* and *n* were initialized from a relatively small value, then gradually increased, and finally the best parameter combination was determined using grid searching. AUC (area under curve) values from the receiver operator characteristic curve (ROC) were compared under different parameter combinations of parameters *r* and *n*, and the results demonstrated that the selection of these two parameters plays an important role in AF/non-AF classification. Small values of parameters *r* and *n* can lead to a better detection accuracy than other selections. The best AUC value for AF detection was 98.15%, and the corresponding parameter combinations for Entropy_AF_ were as follows: *r* = 0.01, *n* = 0.0625, 0.125, 0.25, or 0.5; *r* = 0.05 and *n* = 0.0625, 0.125, or 0.25; and *r* = 0.10 and *n* = 0.0625 or 0.125.

## 1. Introduction

Atrial fibrillation (AF) is a one of the most common arrhythmias, and usually refers to the rapid and irregular fibrillation of the atrium [1]. AF is an important inducement of cardiovascular events such as stroke, heart failure, and sudden death, and has a high morbidity and mortality [2,3]. The incidence of AF is high, and currently ranges from 2% to 4% adults [4]. However, as the symptoms of AF are always brief and weak or even be asymptomatic [5], the diagnosis for AF is relatively difficult, so timely and accurate detection of AF is therefore challenging [6].

The commonly used Holter monitor may miss many cases of paroxysmal AF [7], so the recently developed wearable and long-term electrocardiogram (ECG) monitoring strategies need to have the capability to scan AF and AF-related complications [8,9], which will require a more robust AF detector. There are normally two kinds of approaches for AF scanning—atrial activity analysis-based and ventricular response analysis-based methods [10]. The atrial activity analysis-based method analyses P waves or detects f waves in the ECG data [11,12,13], which requires high quality ECG signals [14]. The ventricular response analysis-based method analyses the irregularity of RR intervals, and has a relatively better tolerance for signal quality, and therefore can be more suitable for AF scanning in the daily environment. Many AF detectors have been proposed in the past few decades, like density histograms [15], Poincaré plot [16], and median absolute deviation [17], as well as various entropy methods [10,18].

Entropy refers to the degree of regularity or irregularity in a time series, and many AF detectors have been proposed based on entropy methods, such as sample entropy (SampEn) [19,20], coefficient of sample entropy (COSEn) [21], and fuzzy measure entropy (FuzzyMEn) [10,22]. All of these methods use the Chebyshev distance to quantify the similarity of two vectors, and thus have some limitations. First, the Chebyshev distance has not been normalized and thus has no upper limit, resulting in uncertainty in the entropy values. Second, the Chebyshev distance only considers the maximum distance between two vectors, ignoring the detailed distance information. To solve these problems, a range function was proposed by Omidvarnia et al. in a new defined range entropy (RangeEn) [23]. In a previous study, we combined the concepts of range function and the advantages of COSEn and FuzzyMEn, and thus proposed an entropy-based AF detector, named Entropy_AF_ [24], which has a better discrimination ability for identifying the AF rhythm from the normal sinus rhythm, for both the MIT-BIH AF database and the clinical wearable AF database.

There are three parameters that need to be initialized in the Entropy_AF_ calculation: (1) embedding dimension *m*, (2) tolerance threshold *r*, and (3) similarity weight *n*. Parameter *m* determines the length of vectors to be compared, parameter *r* is a distance threshold for accepting similar patterns between two vectors, and parameter *n* is a weight for similarity. Parameter *m* depends on the length of time series, and in the current study, as the RR interval time series for the AF rhythm analysis is limited to 30 RR interval segments, parameter *m* was set as 1 according to the previous recommendation [21]. So, how to choose the two remaining parameters *r* and *n* is a problem that needs to be solved urgently. This study addressed the issue and tested the effect on different combinations of the two parameters on the accuracy of detecting the AF rhythm. The test was performed on an open-access MIT-BIH AF database in order to determine the optimal parameter combination.

## 2. Methods

### 2.1. Data

All data used were from the MIT-BIH AF database, which included 25 long-term ECG recordings, with the detailed QRS position and beat annotation files. Each of the recordings was 10 h, and the rhythm annotations were given from the original MIT-BIH AF database, with the expert manual review for verifying four types of rhythm changes: AF (atrial fibrillation), AFL (atrial flutter), J (AV junctional rhythm), and N (used to indicate all other rhythms). Each recording included multiple rhythm segmentations, which are named rhythm episodes herein. The rhythm episodes corresponding to the four rhythm types (AF, AFL, J, and N) were extracted from all 25 recordings, and their number and duration information are shown in Table 1, as well as the corresponding number information of RR intervals. In addition, a 30-beat window length (i.e., 30 RR intervals) was used to segment the rhythm episode without overlap, generating the corresponding RR segments. Table 1 also shows the number of RR segments after the segmenting procedures. From Table 1, it is worth noting that the 30-beat RR segments, whether in AF rhythm and N rhythm (42.6% vs. 54.3%), or in AF rhythm and non-AF rhythm (42.6% vs. 57.4%), reported a nearly 1:1 balanced distribution.

### 2.2. Entropy_AF_ Method

For an RR time series $x\left(i\right)\;\left(1\leq i\leq N\right)$, first form the vector sequences $X_{i}^{m}$ $\left(1\leq i\leq N-m\right)$:

(1) $$X_{i}^{m}=\left\{x\left(i\right),x\left(i+1\right),\cdots ,x\left(i+m-1\right)\right\}$$

where the vector $X_{i}^{m}$ represents m consecutive $x\left(i\right)$.

The distance between vector sequences $X_{i}^{m}$ and $X_{j}^{m}$ is normalized and defined as follows:

(2) $$dX_{i,j}^{m}=d\left[X_{i}^{m},X_{j}^{m}\right]=\frac{\underset{0\leq k\leq m-1}{\max}\left|x\left(i+k\right)-x\left(j+k\right)\right|-\underset{0\leq k\leq m-1}{\min}\left|x\left(i+k\right)-x\left(j+k\right)\right|}{\underset{0\leq k\leq m-1}{\max}\left|x\left(i+k\right)-x\left(j+k\right)\right|+\underset{0\leq k\leq m-1}{\min}\left|x\left(i+k\right)-x\left(j+k\right)\right|+\varepsilon }$$

where ε is a small positive number to avoid the possible denominator of 0. Then, we calculate the similarity degree $DX_{i,j}^{m}\left(n,r\right)$ between the vectors $X_{i}^{m}$ and $X_{j}^{m}$ by a fuzzy function $uX\left(dX_{i,j}^{m},n,r\right)$, defined as follows:

(3) $$DX_{i,j}^{m}\left(n,r\right)=uX\left(dX_{i,j}^{m},n,r\right)=\exp\left(-\frac{{\left(dX_{i,j}^{m}\right)}^{n}}{r}\right)$$

where n is the similarity weight and r is the tolerance threshold.

We define the functions $BX^{m}\left(n,r\right)$ as follows:

(4) $$BX^{m}\left(n,r\right)=\frac{1}{N-m}\overset{N-m}{\underset{i=1}{\sum }}\left(\frac{1}{N-m}\overset{N-m}{\underset{j=1}{\sum }}DX_{i,j}^{m}\left(n,r\right)\right)$$

$BX^{m}\left(n,r\right)$ measures the mean similarity degrees for the vectors at dimension m. Similarly, we define the functions of mean similarity degrees $AX^{m+1}\left(n,r\right)$ for dimension m+1 as follows:

(5) $$AX^{m+1}\left(n,r\right)=\frac{1}{N-m}\overset{N-m}{\underset{i=1}{\sum }}\left(\frac{1}{N-m}\overset{N-m}{\underset{j=1}{\sum }}DX_{i,j}^{m+1}\left(n,r\right)\right)$$

Then, we use a density-based estimation, rather than a probability-based estimation, to generate a quadratic fuzzy AF entropy using the volume of each matching region, i.e., ${\left(2r\right)}^{m}$, as follows:

(6) $$Entropy_{AF}=-\ln\left(\frac{AX^{m+1}\left(n,r\right)/{\left(2r\right)}^{m+1}}{BX^{m}\left(n,r\right)/{\left(2r\right)}^{m}}\right)=-\ln\left(\frac{AX^{m+1}\left(n,r\right)}{BX^{m}\left(n,r\right)}\right)+ln\left(2r\right)$$

We also subtract the natural log of the mean RR interval as follows:

(7) $$Entropy_{AF}=-\ln\left(\frac{AX^{m+1}\left(n,r\right)}{BX^{m}\left(n,r\right)}\right)+ln\left(2r\right)-\ln\left(RR_{mean}\right)$$

where $RR_{mean}$ is the mean of RR intervals in the current RR segment r and $RR_{mean}$ are expressed in units of s.

As shown in Equation (7), directly subtracting the item of $\ln\left(RR_{mean}\right)$ is arbitrary. Lastly, we use a weight to optimize the effect of mean RR interval on the final entropy output, as follows:

(8) $$Entropy_{AF}=-\ln\left(\frac{AX^{m+1}\left(n,r\right)}{BX^{m}\left(n,r\right)}\right)+ln\left(2r\right)-w\times \ln\left(RR_{mean}\right)$$

where w is a weight for optimization, and is set as w=0.8 from our previous study [24].

### 2.3. Parameter Test

Data pre-processing was previously performed on the classified RR episodes. We first segmented the RR intervals into RR segments with a 30-beat window length without overlap. Classification was performed between AF and non-AF rhythm types, the RR segments corresponding to the N, AFL, and J types were merged as non-AF rhythms. The classifier accuracy was assessed via area under curve (AUC) values from the receiver operator characteristic curve (ROC). The ROC curve was a plot of (Se) versus (1−Sp) for many possible values, which varied from the minimum to the maximum of the entropy outputs, with a step of 1% of the range. Here, Se means sensitivity: $Se=\mathrm{TP}/\left(\mathrm{TP}+\mathrm{FN}\right),$ Sp means specificity: $Sp=\mathrm{TN}/\left(\mathrm{TN}+\mathrm{FP}\right)$, where TP, TN, FP, and FN are the numbers of true positives, true negatives, false positives, and false negatives, respectively.

The Entropy_AF_ value was calculated under different parameter settings. The criterion for achieving maximal ROC was used for optimizing the parameters *r* and *n*. In addition, the selection of *m* might depend on the time series length *N* and, as suggested from the previous studies [25,26,27,28], parameters *m* and *N* should meet the requirement of $N\approx 10^{m}\sim 10^{m+1}$. Thus, the embedding dimension *m* was set as 1 due to the short RR segments (30 RR intervals length).

To present the results, histograms of the Entropy_AF_ values were firstly plotted to form a straightforward observation under the representative parameter combinations. Then, the overview of AUC results for classifying AF and non-AF rhythm types was presented. Finally, an inferential analysis of the effect of parameter combination on the entropy output was conducted using an in-depth analysis of the vector distance calculation in Entropy_AF_.

## 3. Results

### 3.1. Classification Results with Different Parameter Combinations

**Figure 1:**
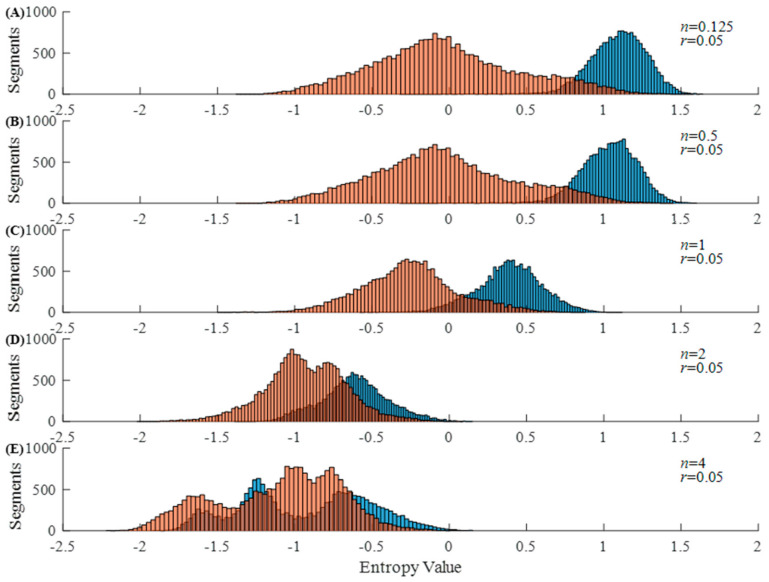
Histogram of Entropy_AF_ values under different parameters: (**A**) *n* = 0.125, *r* = 0.05; (**B**) *n* = 0.5, *r* = 0.05; (**C**) *n* = 1, *r* = 0.05; (**D**) *n* = 2, *r* = 0.05; and (**E**) *n* = 4, *r* = 0.05. The *x*-axis presents the Entropy_AF_ values and the *y*-axis presents the number of 30-beat segments.

**Figure 2:**
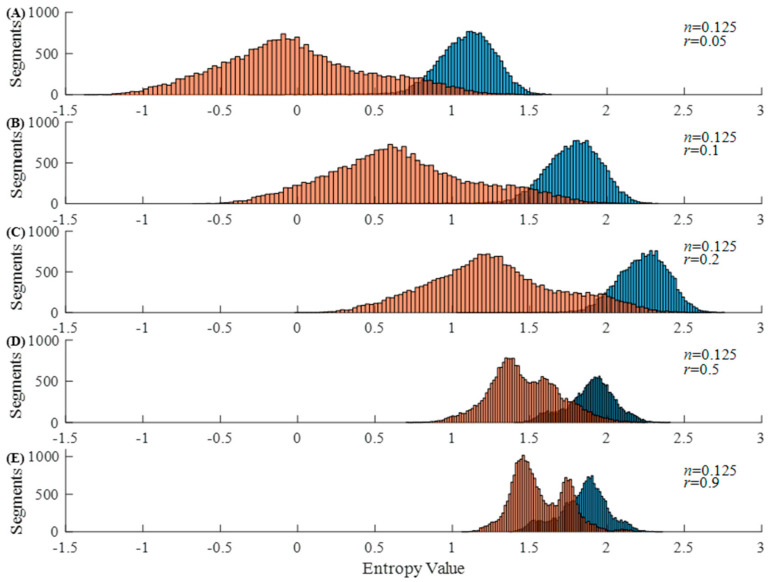
Histogram of Entropy_AF_ values under different parameters: (**A**) *n* = 0.125, *r* = 0.05; (**B**) *n* = 0.125, *r* = 0.1; (**C**) *n* = 0.125, *r* = 0.2; (**D**) *n* = 0.125, *r* = 0.5; and (**E**) *n* = 0.125, *r* = 0.9. The *x*-axis presents the Entropy_AF_ values and the *y*-axis presents the number of 30-beat segments.

Figure 1 and Figure 2 show the histograms of Entropy_AF_ values under different parameter combinations, where blue bars indicate AF and orange bars represent non-AF segments. Figure 1 shows the results when *r* is set as 0.05 and *n* varies from 0.125 to 4. We can observe that the distribution of Entropy_AF_ values for AF and non-AF segments separates differently when using different *n* values. The separation between the two groups with small values (*n* = 0.125 or 0.5) is more visually obvious than that with large values (*n* = 2 or 4), indicating the different classification abilities of Entropy_AF_ measurement when using different parameter combinations. These differences were quantitatively evaluated by the AUC metric as follows: 98.15% for *n* = 0.125, 98.08% for *n* = 0.5, 94.11% for *n* = 1, 82.94% for *n* = 2, and 78.06% for *n* = 4. Figure 2 shows the results when *n* is set as 0.125 and *r* varies from 0.05 to 0.9. We can still observe that the distribution of Entropy_AF_ values for AF and non-AF segments separates differently when using different *r* values. The separation between the two groups with a small value (*r* = 0.05) is still more visually obvious than that with a large value (*r* = 0.9), quantitatively confirmed by the different AUC values, as follows: 98.15% for *r* = 0.05 or 0.1, 97.95% for *r* = 0.2, 93.93% for *r* = 0.5, and 88.27% for *r* = 0.9. These two figures indicate that Entropy_AF_ can distinguish AF from non-AF rhythms better when the parameters of *n* and *r* have small values.

**Figure 3:**
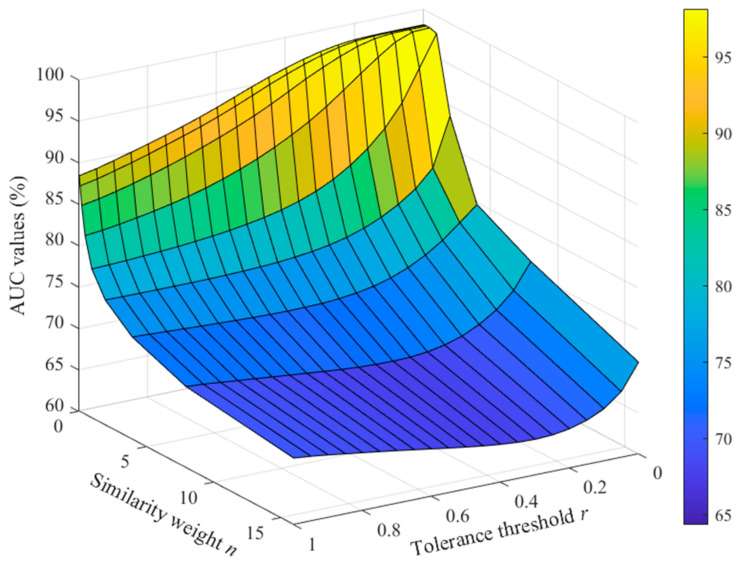
AUC results of the 3D color diagram for the space of scanned *r* and *n*.

Table 2 gives an overview of the AUC results for classifying AF and non-AF rhythm types under different parameter combinations. The tolerance threshold *r* was set from 0.05 to 0.95, with a step of 0.05. In addition, two edge values of *r* = 0.01 and 0.99 were also tested as the optimal AUC value was found on the edge with small *r* and *n* values. The similarity weight *n* was set as $2^{K}$, and *K* varied from −4 to 4, with a step of 1, i.e., *n* had the following values: 0.0625, 0.125, 0.25, 0.5, 1, 2, 4, 8, and 16. Figure 3 shows the AUC results of 3D color diagram for the space of scanned *r* and *n*. The optimal AUC value appeared on the edge, under the condition of the small tolerance threshold *r* and similarity weight *n*. The AUC value had a maximum of 98.15% (marked as bold) when the parameter combinations were set as *r* = 0.01 and *n* varied from 0.0625 to 0.5; *r* = 0.05 and *n* varied from 0.0625 to 0.25; and *r* = 0.10 and *n* varied from 0.0625 to 0.125. When the two parameters increased, the AUC values decreased, which indicates the decreased detection capability for the AF rhythm.

### 3.2. Inferential Analysis from the Calculation of Vector Distances

The distance between vectors was calculated using the ranged function, as shown in Equation (2). As *m* is set as 1, $dX_{i,j}^{m}$ is always 0, and thus the vector similarity degree $DX_{i,j}^{m}\left(n,r\right)$ in Equation (3) and $BX^{m}\left(n,r\right)$ in Equation (4) is always equal to 1. So, the value of Entropy_AF_ only depends on the vector similarity degree when dimension *m* increases to m+1, and the equation is as follows:

(9) $$dX_{i,j}^{m+1}=d\left[X_{i}^{m+1},X_{j}^{m+1}\right]=\frac{\underset{0\leq k\leq m}{\max}\left|x\left(i+k\right)-x\left(j+k\right)\right|-\underset{0\leq k\leq m}{\min}\left|x\left(i+k\right)-x\left(j+k\right)\right|}{\underset{0\leq k\leq m}{\max}\left|x\left(i+k\right)-x\left(j+k\right)\right|+\underset{0\leq k\leq m}{\min}\left|x\left(i+k\right)-x\left(j+k\right)\right|+\varepsilon }$$

**Figure 4:**
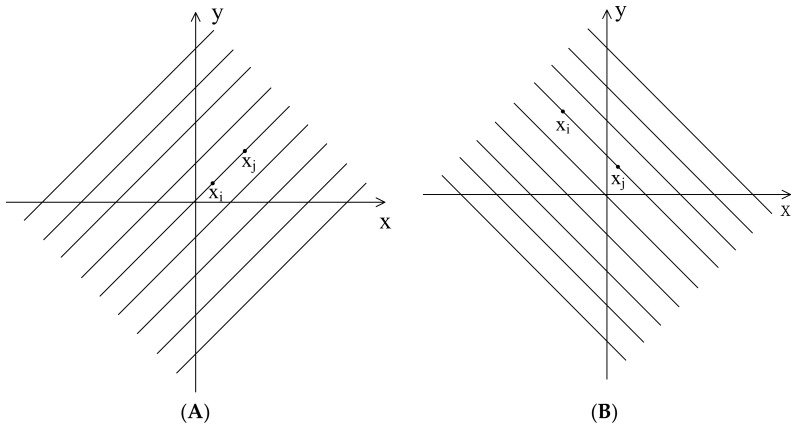
Demonstration of $dX_{i,j}^{m+1}=0$ when two elements are on the lines of (**A**) *y* = *x* + *a* or (**B**) *y* = −*x* + *a*.

**Figure 5:**
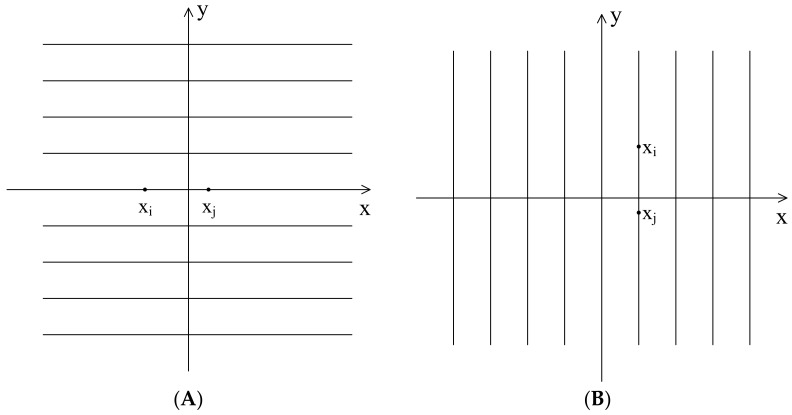
Demonstration of $dX_{i,j}^{m+1}=1$ when two elements are on the lines of (**A**) *y* = *a* or (**B**) *x* = *a*.

**Figure 6:**
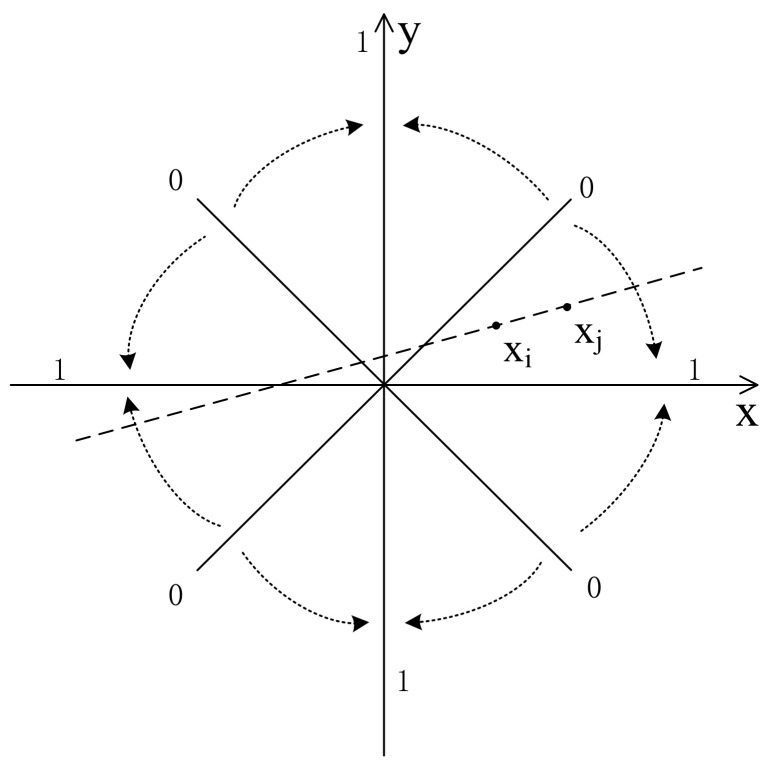
Trend of vector distance with the change of the vector angle.

Herein, each vector $X_{i}^{m+1}$ only includes two consecutive RR intervals. It is important to explore how the change in the two vector elements influences the entropy output. Figure 4, Figure 5 and Figure 6 demonstrate this exploration, where we simply mark these two vectors $X_{i}^{m+1}$ and $X_{j}^{m+1}$ as $x_{i}$ and $x_{j}$, respectively.

From Equation (9), when the two vectors $x_{i}$ and $x_{j}$ are on the lines as shown in Figure 4 (at an angle of 45 degrees to *x*-axis, *y* = *x* + *a* or *y* = −*x* + *a*, where *a* is a constant), $dX_{i,j}^{m+1}$ will be 0. When the two vectors $x_{i}$ and $x_{j}$ are on the lines as shown in Figure 5 (on or parallel to the *x*-axis or *y*-axis, *y* = *a* or *x* = *a*, where *a* is a constant), then $dX_{i,j}^{m+1}$ will be 1. Figure 6 shows the trend of vector distance with the change of vector angle, i.e., when vector angle changes from 45 degrees to 0 degrees with the *x*-axis or *y*-axis, the vector distance changes from 0 to 1.

**Figure 7:**
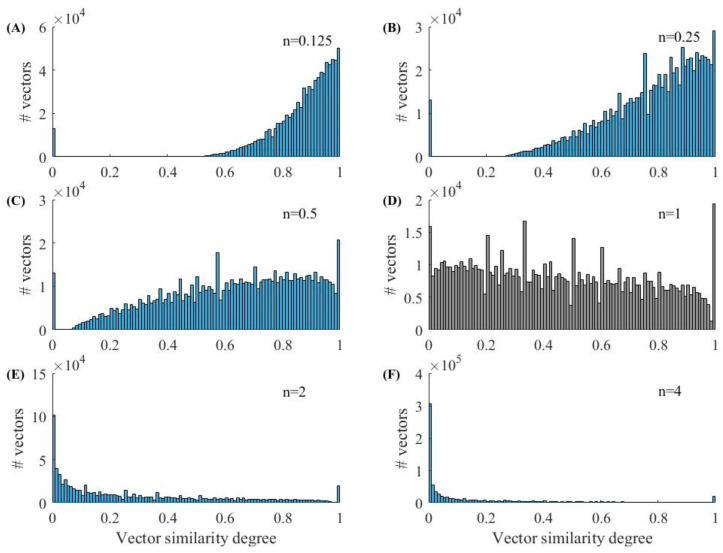
Vector similarity degrees histogram of the AF rhythm. (**A**) *n* = 0.125; (**B**) *n* = 0.25; (**C**) *n* = 0.5; (**D**) *n* = 1; (**E**) *n* = 2 and (**F**) *n* =4.

**Figure 8:**
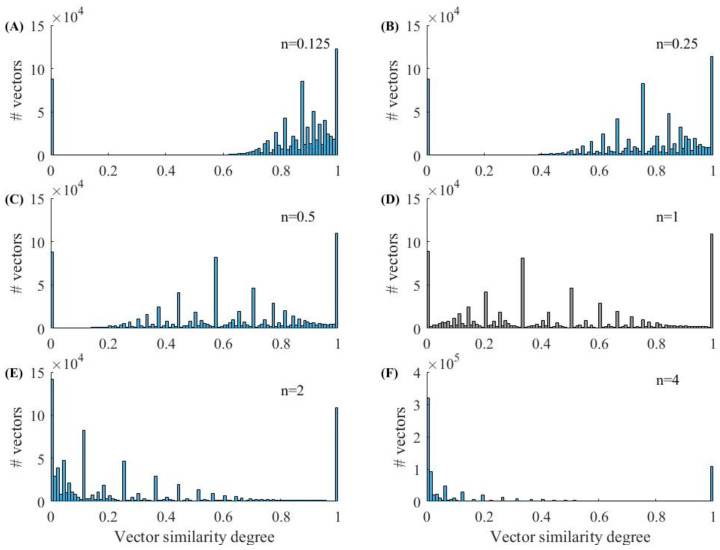
Vector similarity degrees histogram of the N rhythm. (**A**) *n* = 0.125; (**B**) *n* = 0.25; (**C**) *n* = 0.5; (**D**) *n* = 1; (**E**) *n* = 2 and (**F**) *n* =4.

We randomly selected 1000 30-beat segments for the AF and N rhythms each, and further calculated the vector similarity degree $DX_{i,j}^{m}\left(n,r\right)$ as defined in Equation (3). Figure 7 and Figure 8 show the vector similarity degree ($DX_{i,j}^{m+1}$) histograms for AF and N rhythm segments under different parameters of *n*. The raw vector similarity degree distribution could be observed when *n* = 1, as it did not have the effect from the power of *n*, i.e., ${\left(dX_{i,j}^{m+1}\right)}^{n}$. When the power of *n* was employed to the vector similarity degree ($DX_{i,j}^{m+1}$), the distribution of the histogram changed accordingly. As shown in Figure 7 and Figure 8, the raw vector similarity degree ($DX_{i,j}^{m+1}$) had a nearly uniform distribution for both the AF and N rhythms. When applying a small value of power *n* (n<1), the vector similarity degree was enlarged due to the ${\left(dX_{i,j}^{m+1}\right)}^{n}$. So, we can observe the vector similarity degree distribution slope to the right, except for the original vector similarity degree of 0. The opposite situation can be observed when employing a large value of power (n>1).

The entropy value was mainly determined by two factors: (1) the vector similarity degree distribution and (2) the vector similarity judgement rule. From Figure 7 and Figure 8, we can see that a small power of *n* can separate the zero and non-zero vector similarity degree, and then a small threshold for *r* can further easily separate the vectors into similar or unsimilar classes. Thus, we obtained a good performance for the entropy measure to classify AF rhythms. Therefore, we obtained a better classification effect when using a parameter combination of *n* and *r* with small values.

**Figure 9:**
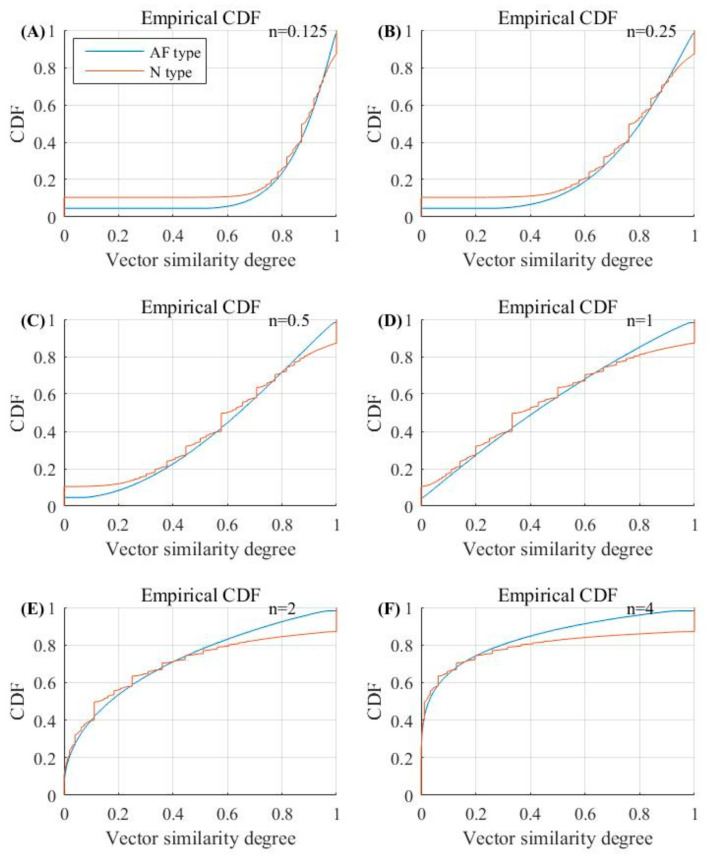
Cumulative distribution function (CDF) for vector similarity degrees. (**A**) *n* = 0.125; (**B**) *n* = 0.25; (**C**) *n* = 0.5; (**D**) *n* = 1; (**E**) *n* = 2 and (**F**) *n* =4.

Figure 9 shows the cumulative distribution function (CDF) for vector similarity degrees for the AF and N rhythm segments when using different parameters of *n*. We can observe that the small parameter of *n* gave more selections for threshold *r* to distinguish the two rhythm types (AF and N). When using *n* = 0.125, the two rhythm types remained in separated curves from each other within a large range of similarity degrees, i.e., a range of 0<r<0.6 could be used to classify the two groups with a high possibility. This inference was confirmed by the quantitative analysis of the AUC metric, as shown in Table 2. When using *n* = 0.125, AUC retained high values of 98.15%, 97.95%, 97.07%, and 95.66% for *r* = 0.1, 0.2, 0.3, and 0.4, respectively. However, when adding parameter *n*, selections of threshold *r* to distinguish the two rhythm types decreased. When using *n* = 0.25, the AUC value decreased to 93.66% for *r* = 0.4. When using *n* = 0.5, the AUC value further decreased to 88.66% for *r* = 0.4.

These differences in the cumulative distribution function of vector similarity degrees could cause differences in entropy values, as shown in Figure 1 and Figure 2. In Figure 1, parameter *r* was set as a fixed value of 0.05, leaving the opportunity to observe the effect of parameter *n* on the entropy outputs. When *n* had s small value of 0.125, 0.25, and 0.5, as shown in Figure 9, the cumulative distribution function of the vector similarity degrees from the two types were different, thus the entropy values were significant different (see Figure 1A−C), demonstrated by the AUC values of 98.15%, 98.15%, and 98.08%, respectively (Table 2). As parameter *n* increased, the value of Entropy_AF_ for the AF and N rhythms both decreased, as shown in Figure 1, sd the increased *n* generated the shrinkage of ${\left(dX_{i,j}^{m}\right)}^{n}$ as shown in Equation (3) and the diminished ${\left(dX_{i,j}^{m}\right)}^{n}$ induced the decrease of entropy values. However, the entropy values in the AF rhythm changed more rapidly than those in the N rhythms. Thus, when *n* increased to 4, the two groups almost merged with each other entirely, resulting in a difficult distinguishment between two groups (see Figure 1E), demonstrated by an AUC value of 78.06% (Table 2).

In Figure 2, parameter *n* remained a fixed value of 0.125, and the parameter *r* increased from 0.05 to 0.9. As demonstrated in Figure 1, the parameter combination of *n* = 0.125 and *r* = 0.05 obtained a high AUC value of 98.15%. When parameter *r* increased, it increased the value of $ln\left(2r\right)$, as shown in Equation (8), and thus generated an increase in Entropy_AF_. In contrast, the value for the non-AF rhythm changed more rapidly than that of the AF rhythms, and when *r* = 0.9, the two groups were merged with a large proportion, resulting in a low AUC value of 88.27%. Therefore, we concluded that when parameters *n* and *r* were both small, the entropy value distributions separated from each other very obviously, and thus Entropy_AF_ had a better capability for AF detection.

## 4. Discussion and Conclusions

This study aimed to analyze the influence of different parameters’ values on Entropy_AF_ for AF identification, and to determine the effective combination for parameters *r* and *n* in order to obtain a good recognition effect.

The determination of entropy parameters plays an important role in the entropy calculation and the physiological signal analysis [25]. Generally, the recommended parameters are as follows: embedding dimension *m* = 1 or 2 according to the length of RR segments, threshold *r* is between 0.1 and 0.25 times the standard deviation (SD) of the time series [26,27], and data length of *N* (varied in different situations) [28,29,30,31]. In this study, *N* was set as 30 and *m* was set as 1, because of the small value of *N*, i.e., as *m* and *N* were suggested to meet the requirement of $N\approx 10^{m}\sim 10^{m+1}$ from the previous studies [25,26,27,28]. We focused on the other two parameters, tolerance threshold *r* and similarity weight *n*, where *n* is the similarity weight in the fuzzy-entropy-based calculation [24,32].

It is worth noting that we used *m* = 1 in the current study due to the short RR segments (30 RR interval length). From the calculation process of entropy, as shown in Equations (1)–(8), *m* = 1 induced the reconstructed vector $X_{i}^{m}$ and only included one element. So, the distances between two vectors, $X_{i}^{m}$ and $X_{j}^{m}$, could not be quantified in Equation (2) and were defined as 0, and the similarity degree $DX_{i,j}^{m}\left(n,r\right)$ was always equal to 1. Thus, the value of Entropy_AF_ was dependent on the vector distances when the embedding dimension increased to *m* + 1 = 2, i.e., from the comparison between two vectors, $X_{i}^{m+1}$ and $X_{j}^{m+1}$. Figure 7 and Figure 8 show the variation trends of the vector similarity degree changed from 0 to 1. When the vector similarity degrees changed to the power of *n*, the similarity degree distribution for both AF and N rhythms also changed. When parameter *n* was small (*n* = 0.125, 0.25, and 0.5), and the smaller *n* was, the large similarity degrees were more concentrated and separated more obviously with a distance of 0. The variation of the similarity degrees led to a change of Entropy_AF_ values for both the AF and N rhythms. When *r* remained a relatively small value (*r* = 0.05), the distribution of entropy value for the AF and N segments separated the most obviously when *n* = 0.125, and when parameter *n* became larger, the two distribution gradually merged with each other. The variation trend was similar to the cumulative distribution function of vector similarity degrees: the two curves for the AF and N segments separated with each other when *n* was small (*n* = 0.125), and as *n* increased, the length of separation from similarity degree 0 for the two curves became shorter. Except for the affection brought from parameter *n*, parameter *r* also had an impact on Entropy_AF_ values. When *n* was set to a constant value (*n* = 0.5), the distribution of entropy values for AF and N segments also changed as *r* increased—from obviously separated (*r* = 0.05) to almost merged with each other (*r* = 0.6).

Another interesting issue is whether the RR interval segments should have been resampled as the cardiac cycle was inherently irregularly spaced in time, and thus generated an unevenly sampled RR interval time series. Usually, spectral estimates of the heart rate variability (HRV) often use an evenly sampled time series to perform the calculation of the frequency analysis method, such as fast Fourier transform (FFT) and wavelet analysis [33]. Clifford and Tarassenko proved that resampling generated an over-estimation of the power spectral density (PSD) and thus decreased the accuracy of the PSD estimation [34]. Kantelhardt et al. also suggested the use of an unevenly sampled RR interval time series, as resampling and the following interpolation could induce artifacts [35]. Thus, although the potential bias exists, the frequency-based method has a need for even sampling in order to perform the implementation of FFT and wavelet analysis. However, in the current study, we did not involve the frequency analysis for RR interval segments, and only focused on the distance calculation between different RR intervals. So, we kept the unevenly sampled RR intervals to avoid the potential bias due to resampling.

The results show that small *r* and *n* values can make the separation of Entropy_AF_ values for the distribution of AF and non-AF segments more obvious, and can improve the detection capability of Entropy_AF_ for the AF rhythm. The best combinations of the two parameters to identify the AF rhythms were as follows: *r* = 0.01, *n* = 0.0625, 0.125, 0.25, or 0.5; *r* = 0.05 and *n* = 0.0625, 0.125, or 0.25; and *r* = 0.10 and *n* = 0.0625 or 0.125.

## Figures and Tables

**Table 1:** MIT-BIH AF database profiles for different rhythm types. For each rhythm type, the numbers and the corresponding percentages (%) are given. (# means the number of).

Variable	AF Rhythm	Non-AF Rhythm			
		N	AFL	J	Total
# rhythm episodes	299 (48.0%)	292 (46.9%)	14 (2.2%)	18 (2.9%)	324 (52.0%)
Total time length (h)	93.5 (37.5%)	149.1 (59.8%)	1.4 (0.6%)	5.2 (2.1%)	155.7 (62.5%)
# RR intervals	521,415 (42.6%)	663,202 (54.2%)	11,710 (1.0%)	26,818 (2.2%)	701,730 (57.4%)
# RR segments	17,247 (42.6%)	21,968 (54.3%)	383 (0.9%)	886 (2.2%)	23,237 (57.4%)

**Table 2:** AUC results for AF detection under different parameter combinations (beat window = 30). The values in bold indicate the highest accuracy.

	*n*	0.0625	0.125	0.25	0.5	1	2	4	8	16
*r*										
0.01		**98.15%**	**98.15%**	**98.15%**	**98.15%**	97.87%	88.78%	79.81%	76.37%	71.06%
0.05		**98.15%**	**98.15%**	**98.15%**	98.08%	94.11%	82.96%	78.06%	73.30%	67.94%
0.10		**98.15%**	**98.15%**	98.12%	97.28%	90.38%	80.86%	76.36%	71.39%	66.30%
0.15		98.13%	98.10%	97.91%	95.89%	87.72%	79.42%	74.99%	70.05%	65.33%
0.20		98.06%	97.95%	97.40%	94.36%	85.72%	78.27%	73.91%	69.07%	64.74%
0.25		97.85%	97.60%	96.64%	92.77%	84.13%	77.38%	73.11%	68.43%	64.42%
0.30		97.47%	97.07%	95.74%	91.22%	82.84%	76.71%	72.52%	68.03%	64.31%
0.35		96.96%	96.41%	94.76%	89.85%	81.83%	76.21%	72.13%	67.81%	64.39%
0.40		96.35%	95.66%	93.66%	88.66%	81.06%	75.86%	71.88%	67.76%	64.57%
0.45		95.68%	94.83%	92.58%	87.59%	80.45%	75.60%	71.73%	67.80%	64.81%
0.50		94.92%	93.93%	91.59%	86.63%	79.96%	75.41%	71.68%	67.89%	65.10%
0.55		94.11%	93.05%	90.69%	85.79%	79.59%	75.29%	71.66%	68.05%	65.40%
0.60		93.31%	92.23%	89.85%	85.04%	79.26%	75.20%	71.68%	68.24%	65.73%
0.65		92.56%	91.46%	89.07%	84.37%	79.01%	75.14%	71.74%	68.45%	66.06%
0.70		91.86%	90.77%	88.34%	83.80%	78.79%	75.10%	71.82%	68.65%	66.38%
0.75		91.21%	90.10%	87.65%	83.31%	78.60%	75.08%	71.91%	68.86%	66.69%
0.80		90.59%	89.45%	87.03%	82.87%	78.44%	75.09%	71.99%	69.07%	66.99%
0.85		90.02%	88.84%	86.43%	82.50%	78.32%	75.09%	72.09%	69.28%	67.28%
0.90		89.45%	88.27%	85.90%	82.14%	78.19%	75.10%	72.20%	69.48%	67.57%
0.95		88.90%	87.92%	85.42%	81.82%	78.11%	75.11%	72.30%	69.69%	67.84%
0.99		88.49%	87.31%	85.06%	81.62%	78.03%	75.14%	72.39%	69.83%	68.05%
